# Fast structural remodeling of *Jatropha curcas* thylakoids upon recovery underpins chloroplast resilience to drought

**DOI:** 10.1007/s00299-026-04019-w

**Published:** 2026-10-26

**Authors:** Richard Hembrom, Helena Sapeta, Ottó Zsiros, Márton Markó, Gergely Nagy, Éva Sárvári, Ádám Solti, M. Margarida Oliveira, Győző Garab, Katalin Solymosi

**Affiliations:** 1 Department of Plant Anatomy, Institute of Biology, Faculty of Science, ELTE Eötvös Loránd University, Budapest, Hungary; 2 Instituto de Tecnologia Química e Biológica, Universidade Nova de Lisboa, Oeiras, Portugal; 3 Present Address: Instituto Nacional de Investigação Agrária e Veterinária, I.P., UEIS Biotecnologia e Recursos Genéticos, Elvas, Portugal; 4 Institute of Plant Biology, HUN-REN Biological Research Centre, Szeged, Hungary; 5 Budapest Neutron Centre, HUN-REN Centre for Energy Research, Budapest, Hungary; 6 Laboratory for Neutron Scattering and Imaging, Paul Scherrer Institute, Villigen, Switzerland; 7 Present Address: Neutron Scattering Division, Oak Ridge National Laboratory, Oak Ridge, TN USA; 8 Department of Plant Physiology and Molecular Plant Biology, Institute of Biology, Faculty of Science, ELTE Eötvös Loránd University, Budapest, Hungary; 9 Department of Physics, Faculty of Science, University of Ostrava, Ostrava, Czech Republic

**Keywords:** Granum structure, Photosynthesis, Photosynthetic apparatus, Rewatering, Small-angle neutron scattering

## Abstract

*Jatropha curcas* is a moderate stem-succulent oil crop renowned for its drought tolerance. While the photosynthetic activity of drought-stressed and rewatered *Jatropha curcas* has been investigated, little is known about the effects of drought and rewatering on chloroplast integrity and thylakoid architecture, critical determinants of photosynthetic performance under stress. Here, we exposed *J. curcas* seedlings to 19 days of water withdrawal followed by rewatering. Leaf relative water content remained unchanged throughout, indicating efficient water retention. Leaf anatomy was largely preserved, but chloroplasts became slightly smaller. Small-angle neutron scattering and transmission electron microscopy revealed a 2–4 nm decrease in granum repeat distance under drought. Electron microscopy further showed that this decrease was stronger in palisade than in spongy parenchyma chloroplasts. In thylakoids, the photosynthetic complexes were adjusted differently under drought: photosystem (PS) I and light harvesting complex (LHC) II remained unchanged, whereas PSII complexes decreased, increasing the PSI/PSII and LHCII/PSII ratios. Among the remaining PSII, larger supercomplexes were proportionally enriched, suggesting their higher drought stability. These changes coincided with a moderate decrease in PSII activity (*F*_*v*_*/F*_*m*_) and increased non-photochemical quenching. After rewatering, granum repeat distances recovered within hours (0.5–1 h in spongy and 5 h in palisade chloroplasts), revealing the involvement of rapidly reversible membrane remodeling under drought, while full recovery of photosynthetic performance and thylakoid protein composition was slower, requiring days. The high plasticity and rapid recovery of the thylakoid architecture is proposed to enable *Jatropha curcas* to restore its drought-affected photosynthetic apparatus and resume photosynthesis once water becomes available.

## Introduction

Global surface temperature is gradually increasing with an average rate of 0.18 °C per decade (Samset et al. 2023). As this warming trend continues and higher temperature coincides with reduced rainfall, drought stress is expected to increasingly impact plant development and growth, both more frequently and over longer periods (Adak et al. 2018; Dietz et al. 2021; Sato et al. 2024). Thus, crop yield losses due to drought are projected to result in total annual revenue losses of 25–30 billion EUR within the EU (Pinke et al. 2024) and 6–8 billion USD in the United States (Xiao et al. 2024), while on a global scale, drought-related economic losses already exceed 307 billion USD per year (Thomas et al. 2024). Drought significantly hinders plant growth by restricting water availability, which is crucial for vital physiological functions such as photosynthesis, nutrient absorption, and the maintenance of cell turgor. Additionally, drought impacts all stages of vegetative development, from seed germination and early seedling growth to the formation of shoots, roots, and the maturation of specialized structures, such as fruits, seeds or tubers (Dietz et al. 2021). Also, early stomatal closure in response to drought further reduces the uptake of CO₂ required for photosynthesis, thereby limiting the energy production essential for growth and development. To avoid these negative effects, several plant species have evolved adaptive strategies to survive in drought-prone environments.

At the vegetative level, adaptation mechanisms include drought avoidance including the development of smaller leaves (George-Jaeggli et al. 2017), the formation of taproots or deep roots (Lindh et al. 2014; Li et al. 2022), the presence of a thick cuticle, stoma crypts or hairy leaf coverings (Nunes et al. 2023), and leaf rolling (Ben-Amar et al. 2020). Succulent features include leaf succulence or large hypodermal water storing cells (Hembrom et al. 2025), while many species of the Euphorbiaceae family, including *Jatropha curcas* exhibit stem succulence (Maes et al. 2009). In addition, *J*. *curcas* tends to express a low level of Crassulacean Acid Metabolism (CAM) in photosynthesis (Winter & Holtum 2015) that can minimize water loss via transpiration during the day by stomata closure because they store CO_2_ taken up during the night in the form of malic acid in the vacuole.

In addition to drought avoidance, several drought tolerance mechanisms have also evolved (Gechev et al. 2012; Mihailova et al. 2022).

*J*. *curcas*, a member of the Euphorbiaceae family, is recognized as a biodiesel plant (Fairless 2007). It is cultivated on a relatively small scale (900,000 hectares globally according to an estimation from 2008) mostly in Asia, Africa and Latin America; and is considered ideal for smallholder farmers as a potential crop for pro-poor development (Brittaine & Lutaladio 2010). Recently, researchers, policymakers and industries have considered this tree for cultivation in arid and semi-arid regions of developing countries to support biofuel production (Castro Gonzáles 2016; Ruatpuia et al. 2024). Due to its significance in biofuel production, high drought tolerance, and ability to grow in barren lands (Fairless 2007; Divakara et al. 2010), numerous studies have focused on developing breeding strategies to enhance seed yield specifically for biofuel applications (Maghuly & Laimer 2013; Riayatsyah et al. 2022; Al-Khayri et al. 2022). Additionally, a few studies have investigated its morphological behavior including leaf growth (Díaz-López et al. 2012; Sapeta et al. 2013, 2016), physiological traits such as photosynthetic activity and stomatal conductance (Arcoverde et al. 2011; Díaz-López et al. 2012; Sapeta et al. 2013, 2016, 2023; Makholwa et al. 2022), biomass production (Achten et al. 2010), oxidative stress responses (Arcoverde et al. 2011) and drought-induced transcriptomic responses (Sapeta et al. 2016). Although light microscopic and ultrastructural studies have been done on *J*. *curcas* chloroplasts under salinity stress (Cao et al. 2023), no research to date has investigated leaf anatomical and chloroplast ultrastructural changes under drought stress and subsequent recovery. As the integrity of chloroplast structure and function is necessary for photosynthetic performance and thus for the biosynthesis of biofuel material, this knowledge is fundamental in the understanding of the background of the drought tolerance of *J. curcas* and can provide information for breeding strategies as well.

In this study, we aimed to understand the correlation between chloroplast ultrastructure, thylakoid composition and photosynthetic activity under drought stress and subsequent recovery. Existing literature presents conflicting data on chloroplast ultrastructural changes during drought stress—some studies report swelling of the intrathylakoidal space (i.e. the thylakoid lumen) (Zhang et al. 2015; 2020; Bai et al. 2016; Shao et al. 2016), while others do not observe significant alterations in plastid structure (Zhu et al. 2021; Mihailova et al. 2022; Hembrom et al. 2025). Similar contradictions were found for the effect of salt stress on chloroplast structure and function, where swelling of the intrathylakoidal space is often reported as a salt stress-related ultrastructural symptom (Yamane et al. 2004, 2008; Ounoki et al. 2023), however, in other experiments functional decline in photosynthesis was accompanied by the decrease in the granum repeat distance (RD) values (Ounoki et al. 2023) or loss of their regularity (Ounoki et al. 2023). Monitoring changes in thylakoid structural and functional integrity are highly relevant in understanding how the structural flexibility and resilience of the photosynthetic membranes contribute to stress tolerance and mitigation processes including repair processes of damaged complexes of the photosynthetic apparatus.

In addition to transmission electron microscopy (TEM), small-angle neutron scattering (SANS) has been proved as a valuable tool for in vivo monitoring of the thylakoid membrane RD variations of grana (Ünnep et al. 2014a, 2020; Ounoki et al. 2023; Hembrom et al. 2025). Therefore, our study focuses on analyzing the structural changes in *J*. *curcas* plastids with TEM and SANS methods as well as by biochemical and spectroscopic analyses after 19 days of drought stress and subsequent recovery upon rewatering the plants. We hypothesized that *J. curcas* responds to drought stress through reversible structural and functional adjustments of its chloroplasts and photosynthetic apparatus, enabling the plant to maintain photosynthetic capacity and recover following rewatering.

## Materials and methods

### Plant material and growth conditions

*Jatropha curcas* L. seeds originally obtained from Cape Verde islands (described in Sapeta et al. 2013) were germinated in clean sand in darkness and grown as described by Sapeta et al. (2013, 2023). After germination, 10-day-old seedlings were transplanted to 3 L pots containing a soil mixture in a ratio of 3:1:1 of sand, peat, and CompoSana quality flower soil [a peat-based soil with perlite and agrosile; pH: 5,9; 2 g L^−1^ salt (KCl); 400 mg L^−1^ N and 330 mg L^−1^ phosphate contents]. The pots were then supplemented with a commercial fertilizer (Osmocote, Scotts, Netherlands, 10.5 g/pot, composed of N:P:K:Mg, 16:9:12:2.5). Plants were grown in the growth chamber for 46 days with the following conditions: a day/night temperature of 28 ± 2 °C/20 ± 5 °C, 35–55% humidity, and a photoperiod of 12 h with a photosynthetic photon flux density (PPFD) of 110–150 μmol photons m^−2^ s^−1^, watering every 2–3 days. Potted plants (46-days-old) were subjected to drought stress by withholding water for 19 days but having the air humidity kept constant as described above (“Dry”) or grown under well-watered conditions (Control; “Ctrl”). At the beginning of the treatments, the first fully developed leaves as counted from the shoot apex were labeled carefully. Following the drought period, “Dry” plants entered a recovery phase with rewatering, an early recovery phase at 0.5 h (“R-0.5 h”), 1 h (“R-1 h”), 3 h (“R-3 h”), 5 h (“R-5 h”) and 24 h (“R-24 h”) as well as prolonged recovery at 3 days (72 h; “R-3d”) and 21 days (“R-21d”), respectively. Control leaves (“Ctrl”) were harvested from well-watered plants at the same time points as the “Dry” leaves (i.e. after 19 days). Additionally, labeled control leaves collected 21 days later, matching the “R-21d” time point, were designated as “Ctrl-R.” Rewatering was achieved by putting the pot of the drought-stressed plant in a container and abundantly watering the soil from the top with approx. 400–500 mL water and then leaving the pot in the container almost full of water for several hours for abundant soaking and efficient rehydration of the almost dry soil, before removing the pot from the container. The same leaves of plants were used first for in vivo activity measurements then were sampled for the different invasive analyses.

### Determination of the leaf and soil relative water contents

The leaf relative water content (RWC) was determined gravimetrically by weighing leaf discs (cut out from the interveinal region) before (fresh weight, FW) and after maintaining them in the dark on wet filter paper under water saturated conditions for 16 h (saturated weight, SW), followed by oven drying at 80 °C to a constant mass (dry weight, DW). RWC was determined using the equation: RWC (%) = 100×(FW – DW) / (SW – DW). Similarly, the soil relative water content (SWC) was determined as described in Hembrom et al. (2025).

### Determination of the leaf chlorophyll content

Chlorophyll (Chl) was extracted from leaf disks (avoiding major leaf veins) or from isolated thylakoids at low light using 80% (v/v) acetone buffered with 5 mM Tricine-KOH (pH 7.8). The extracts were centrifuged at 10,000×g for 5 min (4 °C) and measured by a UV–VIS spectrophotometer (UV-2100, Shimadzu, Tokyo, Japan). Chl and carotenoid contents were calculated according to Porra et al. (1989) and Lichtenthaler (1987), respectively.

### Thylakoid isolation and 2D Blue Native/SDS PAGE

Thylakoid proteomic analyses were carried out according to the protocol available in our laboratory (Sárvári et al. 2022). For isolation of chloroplasts, leaves (without the main veins) were homogenized in isolating buffer [50 mM 4-(2-hydroxyethyl)− 1-piperazineethanesulphonic acid (HEPES)-KOH, pH 7.0, 330 mM sorbitol, 2 mM ethylenediaminetetraacetic acid (EDTA), 2 mM MgCl_2_, 0.1% (w/v) bovine serum albumin, 0.1% (w/v) Na-ascorbate] at 4 °C for 2×3 s by Waring Blender. The homogenate was filtered through four layers of gauze and two layers of Miracloth™ (Calbiochem-Novabiochem, San Diego, CA, USA). Chloroplasts were immediately centrifuged in a swing-out rotor (1000×g, 5 min, 4 °C). The pellet was suspended in a washing buffer [50 mM HEPES–KOH (pH 7.0), 330 mM sorbitol, 2 mM MgCl_2_], and pelleted (1000×g, 5 min, 4 °C). Osmotic shock, chloroplast coupling factor 1 (CF_1_) removal, and storage was according to Sóti et al. (2023).

Thylakoid complexes (500 μg Chl mL^−1^) were separated in 4.5–12.5% Blue Native (BN) gel gradients after solubilization with 1% (w/v) *n*-dodecyl-*β*-D-maltoside (*β*-DM) plus 1% (w/v) digitonin (Sárvári et al. 2022). Polypeptide pattern of the BN bands (applying about 3 mm wide lanes cut out of BN gels to the top of SDS gels) was determined according to Laemmli (1970) but using 10–18% gradient gels containing glycerol (Sárvári et al. 2022). The identity of the complexes was recognized as in Basa et al. (2014). The gels were scanned using an Epson Perfection V750 PRO scanner, and analyzed with Phoretix (Phoretix International, Newcastle-upon-Tyne, UK) software.

### Circular dichroism (CD) spectroscopy

CD spectroscopic measurements were carried out on a J-815 spectropolarimeter (JASCO, Tokyo, Japan). Detached leaves were placed between two glass slides in an optical cell. Spectra were recorded at room temperature between 400 and 800 nm at a scan speed of 100 nm min^−1^, band-pass of 3 nm and step size of 1 nm at room temperature. For each sample, 4–5 scans were averaged. Spectra were normalized to the red-most peak of the absorption spectra, recorded parallel with the CD spectra, and were corrected for baseline distortions. Measurements were repeated on five different leaves. Amplitudes of the psi-type CD bands, at around ( +)505, ( −)675, and ( +)690 nm, were determined using the reference wavelengths of 550, 600, and 750 nm, respectively.

### Chlorophyll-*a* fluorescence induction

Chlorophyll-*a* fluorescence induction was measured by a PAM 101–102-103 Chlorophyll Fluorometer (Walz, Effeltrich, Germany). KL 1500 (Schott, Mainz, Germany) light sources were applied. Prior to the measurements, leaves were dark adapted for 30 min and then illuminated with far-red light for 3 s in order to eliminate reduced electron carriers (Belkhodja et al. 1998). A modulation frequency of 1.6 kHz and a PPFD of less than 1 µmol photons m^−2^ s^−1^ was used for the measuring light to determine *F*_*o*_, the minimum fluorescence level. The maximum fluorescence yield *F*_*m*_ was measured by the application of a 0.7 s pulse of saturating white light (PPFD of 3,500 µmol photons m^−2^ s^−1^). The variable fluorescence was determined as *F*_*v*_ = *F*_*m*_ – *F*_*o*_. Actinic white light (PPFD of 100 µmol photons m^−2^ s^−1^), together with a measuring light of 100 kHz frequency was provided for light acclimation; *F*_*m*_*’* was determined as the fluorescence yield in response to a saturating pulse (PPFD of 3,500 µmol photons m^−2^ s^−1^). Steady-state status was confirmed when no change was found in maximal fluorescence values (*F*_*m*_*’*) between two white light flashes separated by 100 s. The non-photochemical quenching (NPQ) (Baker & Rosenqvist 2004) was calculated as *NPQ* = (*F*_*m*_-*F*_*m*_*’*)/*F*_*m*_*’*.

### Light and transmission electron microscopy

Small, 2 mm ×1 mm leaf pieces from leaf regions located next to the regions used for SANS measurements were fixed in 2.5% glutaraldehyde in 70 mM Na_2_HPO_4_-KH_2_PO_4_ buffer (pH 7.2), and kept for 1 week at 4 °C. After rinsing with the same buffer three times for 15 min, post-fixation with 1% osmium tetroxide dissolved in the same buffer for 2 h, another rinsing with the buffer three times for 15 min, dehydration in ascending ethanol series, and embedding after proper orientation into Durcupan ACM resin (Fluka Chemie AG, Switzerland), the resin was polymerized at 60 °C for 72 h. Ultrathin Sects. (70 nm) were cut from the block using a Reichert Jung Ultracut-E ultramicrotome (Reichert Jung AG, Vienna, Austria) and were stained with uranyl acetate and Reynold’s lead citrate. Transmission electron microscopic (TEM) analyses were carried out with a JEOL JEM 1011 (JEOL Ltd., Tokyo, Japan) at 80 kV accelerating voltage. Digital images were taken using Olympus Morada CCD camera (Olympus Optical Co. Ltd., Tokyo, Japan). ImageJ software (National Institutes of Health) was used to calculate the repeat distance values by performing Fast Fourier Transformation (FFT) on the selected regions (i.e. grana) of the micrographs. In addition, ImageJ was also used to measure granum diameter and height (in nm).

The same blocks were used to cut semi-thin sections, which were then stained with toluidine blue and observed with an Olympus BH2-RFCA light microscope (Olympus, Tokyo, Japan) equipped with Nikon COOLPIX 950 (Nikon, Tokyo, Japan) digital camera. Light microscopic sections were used to get a clear picture of the leaf structure, and thus to identify spongy and palisade parenchyma cells also on the TEM slides.

### Small-angle neutron scattering measurements and data treatments

The experiment was performed on the SANS II instrument of the Paul Scherrer Institute (Villigen, Switzerland). Measurements were performed on samples in air, without cuvettes or surrounding water or D_2_O and without any pretreatment and infiltration (free standing measurements). To increase the signal-to-noise ratio, 3 overlapping leaf segments were placed into the neutron beam. As a subtractable background, we measured the air scattering. The measurements were performed at the following settings: sample-to-detector (SD) distance, 6 m; collimation, 6 m; wavelength, 5.52 Å. The detector efficiency calibration-related measurements were performed on H_2_O, empty cuvette and cadmium, with instrument settings of: SD, 1.5 m; collimation, 2 m; wavelength, 5.52 Å.

For data treatment the “Graphical Reduction and Analysis SANS Program” package (GRASP) was used (Dewhurst 2023). The observed 2D scattering patterns did not reveal significant anisotropy. Thus, the analysis was performed on 360 degree radially averaged scattering curves. Data are presented without absolute scaling due to uncertainty of and variation in the total thickness of the measured multiple leaf segments.

The scattering curves exhibited a characteristic peak around 0.03 Å^−1^, corresponding to the Bragg-peak arising from the periodic thylakoid membranes in the leaf segments. To quantify the structural changes observed with SANS, the scattering curves were fitted with the sum of a power function and a Gaussian. The fitting range was 0.0184 Å^−1^ – 0.0444 Å^−1^.

Complementary SANS experiments were performed on an undetached leaf of a live plant at the extended Q-range small-angle neutron scattering diffractometer (EQ-SANS) at the Spallation Neutron Source (SNS) of the Oak Ridge National Laboratory (ORNL) (Heller et al. 2018). The intact plant (with soil and pot) was placed in the sample environment area. The leaf was set close to perpendicular to the neutron beam. We were able to study 3 non-overlapping areas of the leaf with the help of the positioning of the sample table. The instrument was set to the 30 Hz (frame-skipping) mode with 4 m sample-to-detector distance and 0.25–0.614 nm and 0.978–1.342 nm wavelength bands. This provided information on a momentum transfer range of 0.0615–4.35 nm^−1^. Data reduction was performed via the drtsans software (Heller et al. 2022).

### Statistics

Experiments were repeated independently 3 times, with 2–3 biological replicates in each experiment, the number of repetitions of the different parameters are given in the corresponding figure legends. Statistical analyses were performed using GraphPad Prism 9 software (USA). Brown-Forsythe and Welch ANOVA were applied for the samples with normal distribution and unequal standard deviations, followed by Dunnett’s T3 multiple comparisons test. When equal standard deviations were observed in samples having normal distribution, ordinary one-way ANOVA was used, followed by Tukey’s multiple comparisons test. When the data did not pass the normality test, the Kruskal–Wallis ANOVA test was used, followed by Dunn’s multiple comparisons test.

For CD, mean and standard error values were provided for at least 5 independent repetitions, respectively. The datasets were compared between samples by one-way ANOVA using OriginPro 8 for Windows. All statistical differences were considered significant at *P* ≤ 0.05.

## Results

### Relative water contents of the soil and the leaves

The soil relative water content (SWC) decreased significantly during the water withholding treatment, dropping from approx. 89% to 12%, and being fully recovered to control levels within 3 days after rewatering (to approx. 90%) (Table 1). Considering that the drought-stressed plants showed wilting of the leaf petioles, a visible symptom of drought stress which was restored quickly after rewatering, it is interesting that the RWC of the leaf mesophyll regions did not change significantly during drought stress and subsequent recovery (Table 1). In parallel with our SANS and TEM analyses, we also determined leaf RWC after 0.5 h (89.2±0.7%), 1 h (88.4±0.4%), 3 h (90.1±2.4%), 12 h (92.6±0.5%) and 24 h (94.3±0.7%), but also did not observe any significant change during these first phases of rewatering.

**Table 1:** Changes in the soil and leaf relative water contents (SWC and RWC, respectively), and photosynthetic pigments such as chlorophyll (Chl) *a* and b, and carotenoids (Car) of the leaves of drought-stressed and rewatered *J*. *curcas* L. plants

Treatment	Ctrl	Dry	R-3d	R-21d	Ctrl-R
SWC (%) (*n* = 4–7)	89.4 ± 3.1^a^	11.85 ± 1.4^b^	90.45 ± 4.4^a^	86.9 ± 4.6^ab^	90.26 ± 2.2^a^
Leaf RWC (%) (*n* = 5–26)	92.2 ± 1.2^abc^	89.8 ± 0.7^ac^	86.6 ± 2.2^a^	95.3 ± 0.9^b^	94.5 ± 2.1^bc^
Chl *a* (*n* = 6–10)	13.19 ± 0.91^ab^	11.41 ± 0.38^a^	12.80 ± 0.38^ab^	13.34 ± 0.37^ab^	15.52 ± 0.70^b^
Chl b (*n* = 6–10)	3.80 ± 0.28^a^	3.76 ± 0.16^a^	4.19 ± 0.12^a^	3.91 ± 0.15^a^	4.51 ± 0.19^a^
Chl *a*+b (mg/g DW) (*n* = 6–10)	17.00 ± 1.19^ab^	15.17 ± 0.53^a^	17.00 ± 0.48^ab^	17.25 ± 0.51^ab^	20.03 ± 0.89^b^
Chl *a*/b (*n* = 6–10)	3.48 ± 0.03^a^	3.05 ± 0.07^b^	3.06 ± 0.06^bc^	3.42 ± 0.06^ac^	3.44 ± 0.04^a^
Car/Chl (*n* = 3–6)	0.22 ± 0.01^a^	0.22 ± 0.01^a^	0.21 ± 0.00^a^	0.20 ± 0.00^a^	0.20 ± 0.00^a^

Abbreviations refer to the following samples: “Ctrl”––control plants analyzed at the same time as “Dry” samples; “Dry”––19-day drought-stressed plants; “R-3d” and “R-21d”––plants sampled 3 days and 21 days after rewatering, respectively; “Ctrl-R”––control plants analyzed at the same time as “R-21d.” Means and standard error values are provided. Significant differences were calculated using ordinary one-way (Car/Chl) and Kruskal–Wallis (RWC, SWC, Chl *a*, Chl b) ANOVA tests followed by Tukey’s and Dunn’s multiple comparison tests, respectively. For the remaining data (Chl *a*+b, Chl *a*/b), the Brown-Forsythe and Welch ANOVA test was performed, followed by Dunnett’s multiple comparison post hoc test. Significant differences are indicated by different letters (*P* ≤ 0.05, *n* = 3–26)

### Photosynthetic pigment contents

Our treatments did not induce any significant changes in the Chl content of the leaves as well as in the carotenoid to Chl ratio (Car/Chl) (Table 1). However, older control leaves (Ctrl-R) had significantly higher Chl content than other samples. The Chl *a*/b ratio of leaves decreased during drying, and the recovery was not full after 3 days, but only after 21 days (Table 1).

### Photosynthetic activity of PSII

**Fig. 1:**
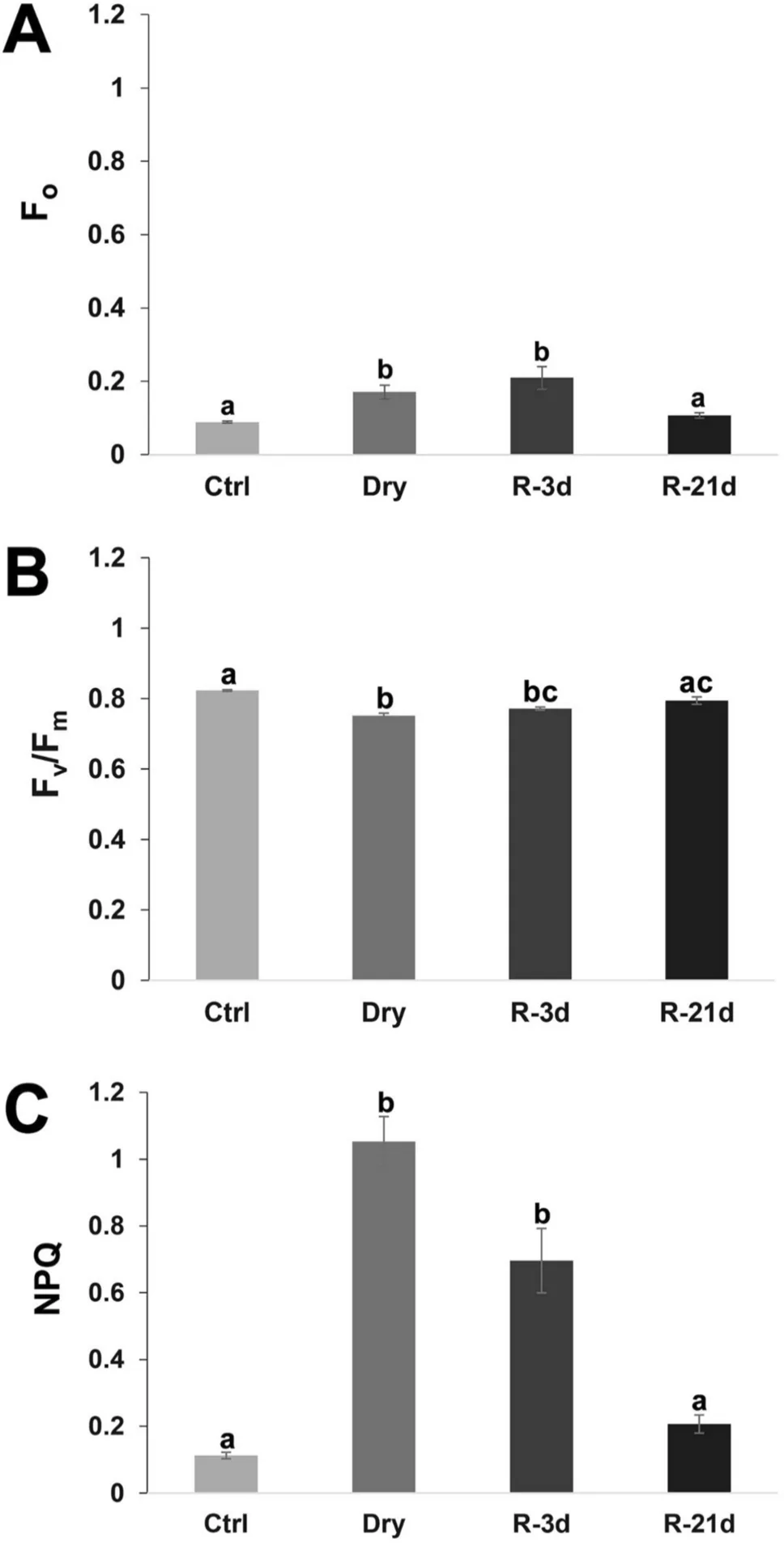
Changes in the excitation energy allocation in the leaves of drought-stressed and rewatered *J*. *curcas* L. plants, as inferred from chlorophyll-*a* fluorescence transients. “Ctrl”––control plants analyzed at the same time as “Dry” samples; “Dry”––19-day drought-stressed plants; “R-3d” and “R-21d”––plants sampled 3 days and 21 days after rewatering, respectively. **A** *F*_*o*_ – minimum fluorescence intensity of a sample in its resting or dark-adapted state; **B** *F*_*v*_*/F*_*m*_ – parameter reflecting the photochemical activity and structural dynamics of PSII; **C** *NPQ* – non-photochemical quenching. Means and standard errors are provided. Significant differences for *F*_*v*_*/F*_*m*_ were calculated using Brown-Forsythe and Welch ANOVA followed by Dunnett’s multiple comparisons test. For the remaining data, the Kruskal–Wallis ANOVA test was used, followed by Dunn’s multiple comparisons test. Significant differences are indicated by different letters (*P* ≤ 0.05, *n* = 7–27)

To characterize the photosynthetic apparatus functionally, Chl-*a* fluorescence induction kinetic measurements were performed. We determined the *F*_*o*_ and *F*_*m*_ values, and *F*_*v*_/*F*_*m*_ ratios, which refer to the minimum and maximum fluorescence levels and a ratio of the variable fluorescence (*F*_*v*_=*F*_*m*_−*F*_*o*_), and the maximum fluorescence, respectively. The value of this latter ratio depends on the photochemical activity and structural dynamics of PSII reaction centers (Sipka et al. 2021; Garab et al. 2023). After 19 days of drought treatment, the *F*_*o*_ value increased (Fig. 1A), and the *F*_*v*_*/F*_*m*_ ratio decreased moderately but significantly (Fig. 1B). Both the *F*_*o*_ level and the *F*_*v*_*/F*_*m*_ ratio recovered fully only after 21 days of rewatering. We also determined the *NPQ* levels, which increased notably during drought stress and started to decline after rewatering being, reaching control level after 21 days of recovery (Fig. 1C).

### Analyses of the chiral macro-arrays of the pigment–protein complexes

**Fig. 2:**
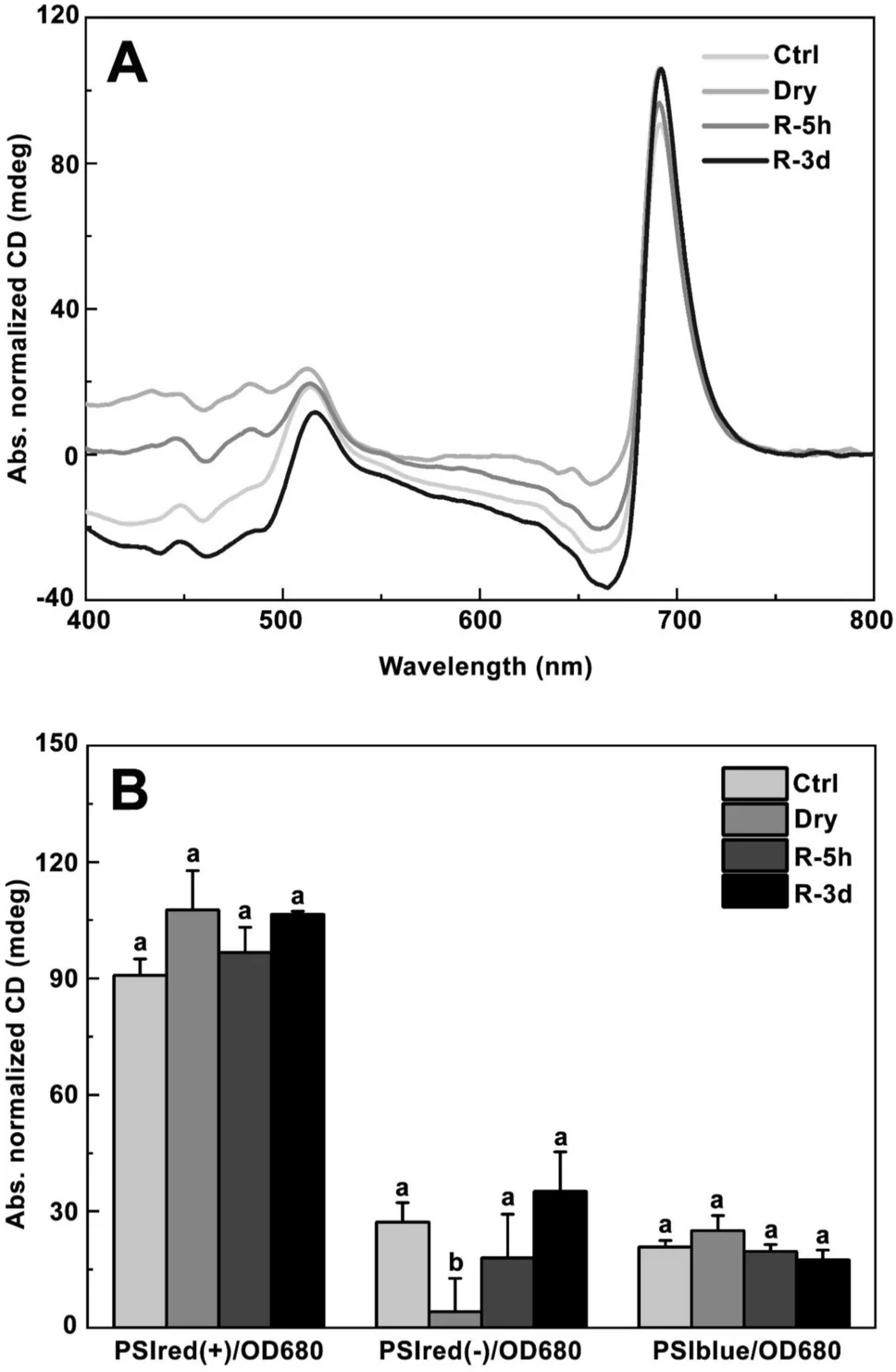
Circular dichroism (CD) spectra of the leaves of drought-stressed and rewatered *J*. *curcas* plants. **A** Typical CD spectra of *J. curcas* leaf segments of control (“Ctrl”––analyzed at the same time as “Dry” samples), 19-day drought-stressed (“Dry”) and subsequently rewatered plants (“R-5 h” and “R-3d” represent plants after 5 h or 3 days after rewatering, respectively) and **B** the means and standard error values of the amplitudes of the psi-type bands, ( +)CD_690-750_, (−)CD_675-600_, and ( +)CD_505-550_ in them. The spectra were normalized to the absorbance of the leaves at around 685 nm, relative to that measured at 750 nm. Different letters in panel B indicate statistically significant differences according to ANOVA followed by Bonferroni post-hoc test (*P* ≤ 0.05, *n* = 5)

As shown in Fig. 2A, the control leaves display intense psi-type CD bands, which originate from chiral macro-arrays of the pigment–protein complexes. The spectra also contain weak excitonic bands at around 650 nm and in the Soret region. Drought stress significantly diminished the (−) 675 nm band (Fig. 2 A, B). Evaluation of the changes in the much weaker excitonic bands in the Soret region is hindered by possible light scattering artifacts, which are more intense in the blue than in the red spectral region (Garab 1996). Rewatering restored the (−) 675 nm band leaving the ( +) 690 and ( +) 505 nm bands essentially unchanged.

### Proteomic analysis of the thylakoid membranes

**Fig. 3:**
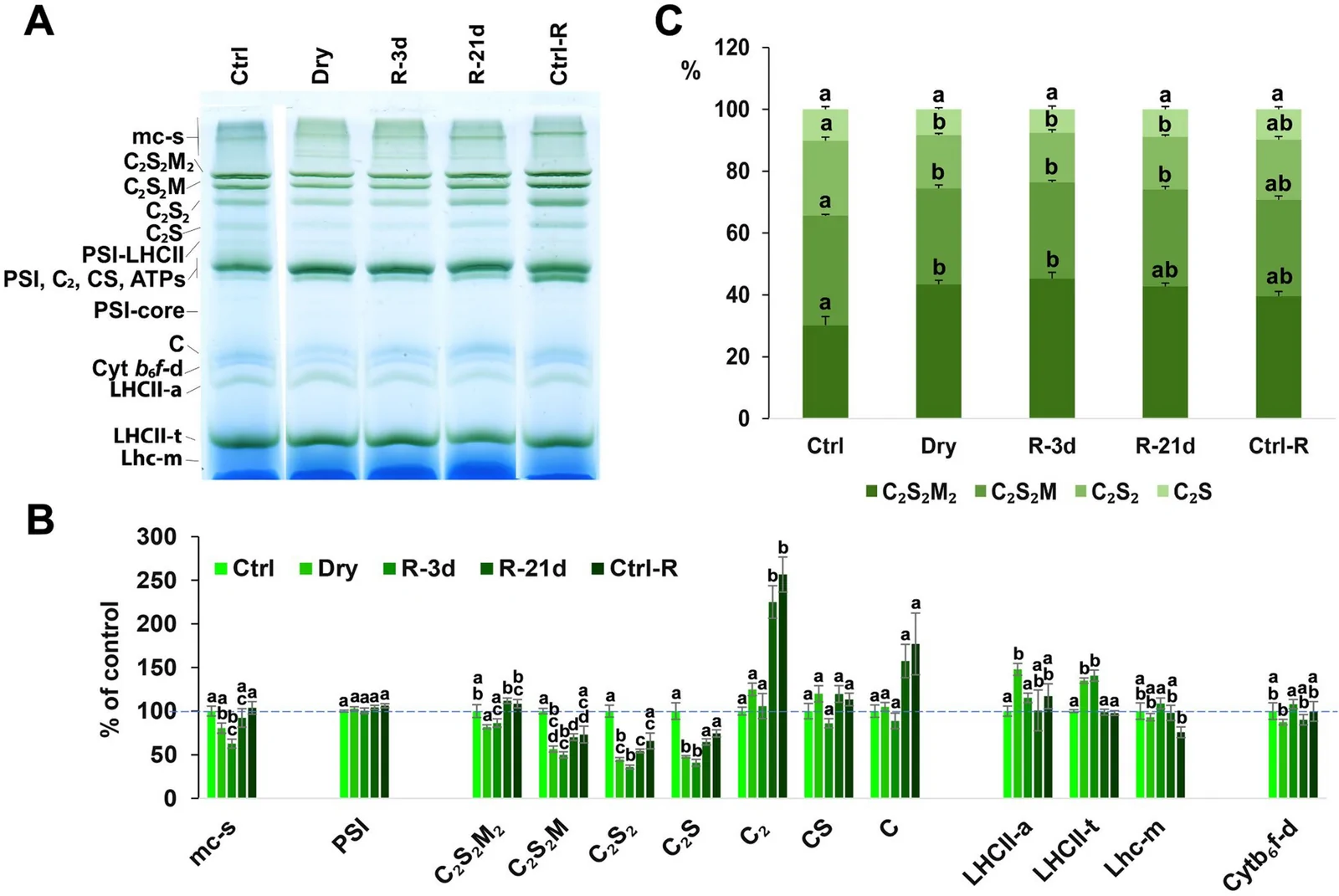
Blue Native PAGE of thylakoid complexes isolated from the leaves of drought-stressed and rewatered *J*. *curcas* plants. “Ctrl”––control plants analyzed at the same time as “Dry” samples; “Dry”––19-day drought-stressed plants; “R-3d” and “R-21d”––plants sampled 3 days and 21 days after rewatering, respectively; “Ctrl-R”––control plants analyzed at the same time as “R-21d.” **A** BN patterns of thylakoid complexes. Thylakoids (500 µg Chl mL^−1^) were solubilized using 1% *β*-DM plus 1% digitonin and separated in 4.5–12.5% gel gradient. **B** Changes in the distribution of complexes during treatments. The amounts of complexes (in pixel density) are given as the percentage of control values. **C** Percentage distribution of the PSII supercomplexes in the differently treated samples. *PS *photosystem, *C *PSII core monomer, *C*_*2*_ PSII core dimer, *LHC* (Lhc) light-harvesting complex, *S* *M *strongly, and moderately attached LHCII trimers in PSII supercomplexes, respectively CP––chlorophyll-protein; LHCII-a––LHCII-assembly: CP29-CP24-LHCII-t; Cyt *b*_*6*_*f*––cytochrome *b*_*6*_*f* complex; ATPs––ATP synthase; mc––megacomplex; t––trimer; d––dimer; m––monomer. **B**, **C** Means and standard error values are provided. Significant differences were calculated by Brown–Forsythe and Welch ANOVA followed by Dunnett’s multiple comparison post hoc test. Significant differences are indicated by different letters (*P *≤ 0.05, *n* = 5–13)

Studying the complex composition of thylakoids by BN PAGE, 11 clear bands were detected in addition to megacomplexes (mc-s) and Lhc monomer (Lhc-m) (Fig. 3A). These bands were identified as different organizational forms of photosystems (PSs) PSI and PSII, light harvesting complexes (LHCs, Lhcs), cytochrome *b*_*6*_*f* (Cyt *b*_*6*_/*f*), and ATP synthase complexes based on their polypeptide patterns (see Fig. S1).

Comparing the distribution of thylakoid complexes to that of the control samples, the proportions of PSI, CS, C and Cyt *b*_*6*_*f* complexes were unchanged during the treatments (Fig. 3B). However, a decrease in the relative amount of PSII, mainly that of the supercomplex forms (PSII-s: C_2_S_2_M, C_2_S_2_, C_2_S), was shown under drought. Considering the significantly decreased absolute amounts of PSII complexes (in mg Chl g^−1^ DW) and the fact that PSII-s’ disassembly hardly increased the amounts of the small PSII complexes (C_2_, CS, and C, Figs. 3B and S2), a fraction of PSII must have degraded. This was also supported by the elevation of the PSI/PSII and LHCII/PSII ratios under drought stress (Fig. S3A-B). Even though drought resulted in a decrease of PSII-s pool overall, the PSII-s that remained were shifted toward the larger supercomplex forms. In other words, the total pool of PSII-s got smaller, but the C_2_S_2_M_2_, C_2_S_2_M types made up a larger share within that reduced pool and therefore appeared more prominent in the PSII supercomplexes distribution plots (Figs. 3A, C, S3C). PSI was mainly present without binding LHCII-trimer (see Fig. S2). Regarding the LHCII complexes, the relative amounts of LHCII assemblies increased, and the absolute and relative amounts of the free LHCII trimers increased during drought (Figs. 3B, S2) causing proportional changes in the organizational forms of LHCII (Fig. S3D). This increase in the free LHCII pool might originate from the disassembled PSII supercomplexes. Most of these drought-driven changes did not fully recover within 3 days of rewatering, implying de novo synthesis to repair damage rather than a quick reversible adjustment.

### Leaf anatomy

**Fig. 4:**
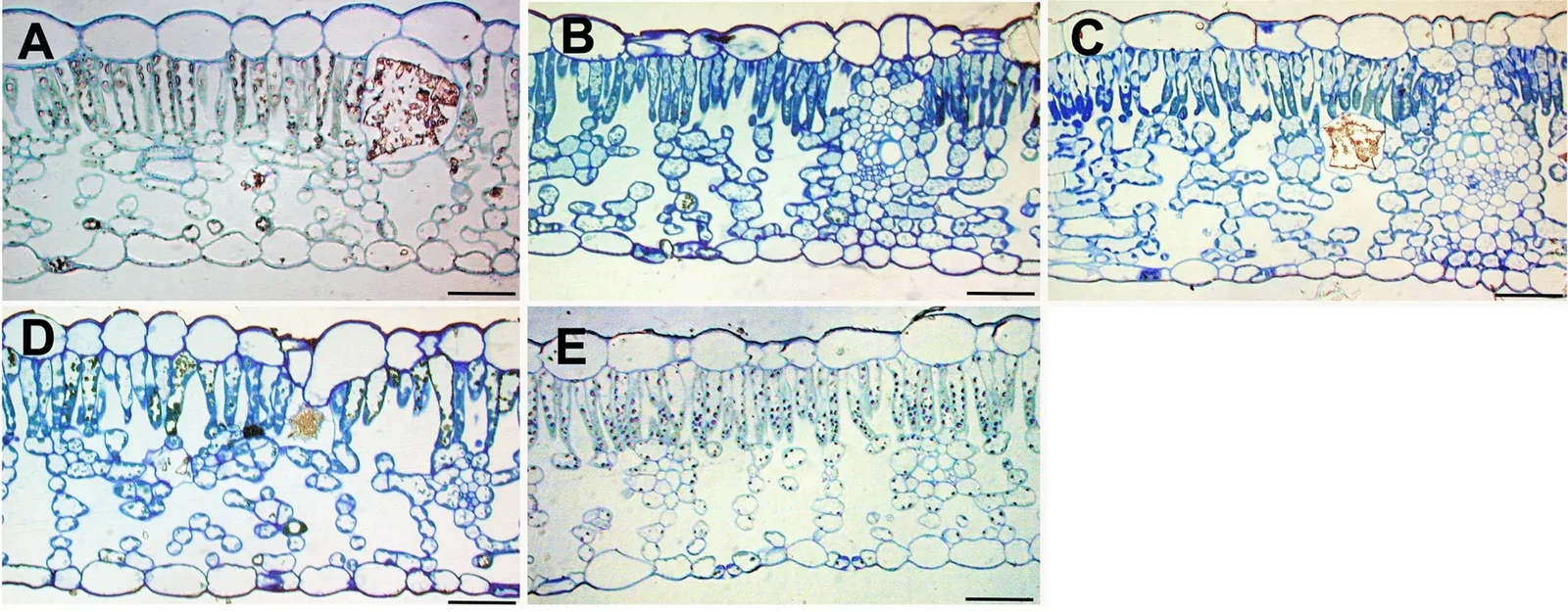
Light micrographs of the leaves of drought-stressed and rewatered *J*. *curcas* plants. **A** Control, well-watered plants (“Ctrl,” analyzed at the same time as “Dry” samples); **B** 19-day drought-stressed plant (“Dry”); **C**–**F** plants rewatered for 1 h (C–– “R-1 h”), 5 h (D–– “R-5 h”) and 3 days (E, 72 h, “R-3d”) after drought stress, respectively. Scale bar: 50 μm

The first mature leaves of the control (46-day-old) *J. curcas* plants were amphistomatic, i.e. presenting stomata on both the adaxial and abaxial epidermis layers (Fig. 4). The mesophyll of the leaves was heterogeneous, the dorsiventral leaves had one cell-layered palisade parenchyma at the adaxial leaf side, and spongy parenchyma located at the abaxial leaf side. Idioblasts containing druse crystals were often present in the mesophyll. We did not observe large visible changes in the leaf anatomical and tissue features associated with drought stress.

Smaller and larger veins were often observed in the samples (Fig. 4B, C), separating distinct regions of the leaf, i.e. the vein islets. While large intercellular air spaces were present within the vein islets, the neighboring islets were fully separated from each other by the tissues located at the veins which span across the entire leaf width (Fig. 4).

### Chloroplast ultrastructure

**Fig. 5:**
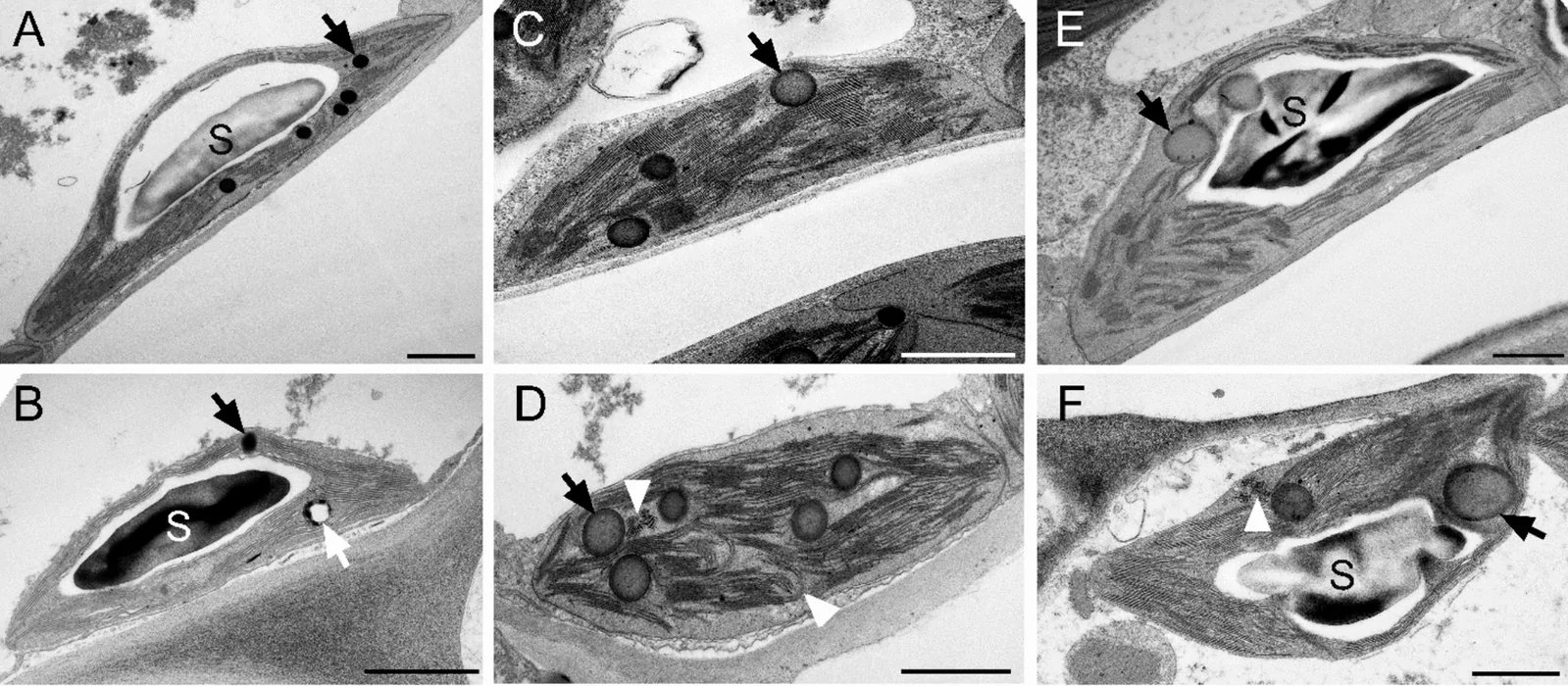
Transmission electron micrographs of leaf chloroplasts in palisade **A**, **C**, **E** or spongy **B**, **D**, **F** parenchyma cells of drought-stressed and rewatered *J*. *curcas* plants. Control plant **A**, **B**; “Ctrl), 19-days drought-stressed plant **C**, **D**; “Dry”, and 19-days drought-stressed and subsequently rewatered plant, 3 days (72 h) after rewatering **E**,** F**; “R-3d”. Scale bar: 1 µm. S: starch, black arrow: plastoglobuli, black arrowhead: curved thylakoid membrane, white arrow: phenolic inclusion, white arrowhead: ferritin

Chloroplasts of control plants showed regular ultrastructure, with grana and stroma thylakoids (Fig. 5A-B) both in palisade (Fig. 5A) and spongy (Fig. 5B) parenchyma cells. Large biological variability was observed in the size and electron density of the plastoglobuli among different plants, i.e. in the leaves of some control plants, electron dense plastoglobuli, while in others large, electron transparent plastoglobuli were present (not shown). Similar variability was found among the plastoglobuli found in the leaves of different drought-stressed and different recovered plants, therefore, in our ultrastructural analyses we focused on chloroplast size and thylakoid structure. Occasionally, we detected starch in the control plastids, vesicle clusters or tubuli below the envelope membrane (probably representing the peripheral reticulum) (not shown), as well as phenolic inclusions (Fig. 5B) and ferritin in the plastid stroma (Fig. 5F). In case of chloroplasts located in the spongy parenchyma cells, slight swelling of the stroma thylakoids was often visible (Fig. 5B).

We have analyzed the leaves of four drought-stressed plants. Overall, drought-stressed and control leaves’ chloroplasts exhibited similar general ultrastructure (e.g. no major stress damage was observed), although starch grains were absent from the chloroplasts of all four drought-stressed plants.

We have analyzed in detail the alterations of the thylakoid structure upon recovery from drought stress. For this, we collected samples from plants after 1 h, 5 h, and 3 days (72 h) of rewatering (Fig. 5E,F). Interestingly, except for the gradual reappearance of starch grains, no characteristic structural differences were observed in the plastid and thylakoid structure during recovery from drought. This indicates the structural stability of the plastids of this plant toward drought stress and recovery.

Quantitative analyses of light microscopic and TEM data showed that the size of chloroplasts decreased significantly under drought (Table 2). Both the average length and width of chloroplasts decreased significantly by approx. 0.8–0.9 µm, however, as the width of these lens-shaped organelles was already smaller, this resulted in significantly higher chloroplast length-to-width ratios in the stressed plants (Table 2). During rewatering for 1 and 5 h, we observed restoration tendencies in the chloroplast length and width values. However, after 3 days of recovery, the size of the plastids significantly decreased, with very small and rounded (small length/width ratio) plastids appearing in the samples (Table 2). The latter morphological changes may be associated either with chloroplast division or also with the reaccumulation of starch grains within the plastids.

**Table 2:** Morphological parameters of chloroplasts and granum repeat distance (RD) values in the leaves of drought-stressed and rewatered *J*. *curcas* plants analyzed using light microscopy (chloroplast length and width in μm, and their ratio) and transmission electron microscopy (granum repeat distance in nanometers)

	Ctrl	Dry	R-1 h	R-5 h	R-3d
Chloroplast length (µm)	4.98 ± 0.11^a^ (*n* = 80)	4.09 ± 0.06^b^ (*n* = 105)	4.59 ± 0.10^c^ (*n* = 63)	4.54 ± 0.09^c^ (*n *= 72)	2.16 ± 0.04^d^ (*n* = 83)
Chloroplast width (µm)	2.49 ± 0.08^ab^ (*n* =80)	1.69 ± 0.04^c^ (*n* = 105)	1.99 ± 0.06^ad^ (*n* = 63)	1.98 ± 0.05^bd^ (*n* = 72)	1.51 ± 0.03^e^ (*n* = 83)
Chloroplast length/width	2.16 ± 0.09^a^ (*n* =80)	2.58 ± 0.08^b^ (*n* =105)	2.42 ± 0.08^ab^ (*n *= 63)	2.38 ± 0.07^ab^ (*n *= 72)	1.48 ± 0.04^c^ (*n* = 83)
Granum repeat distance (entire mesophyll) (nm)	19.65 ± 0.11^a^ (*n* =739)	16.18 ± 0.08^b^ (*n* =914)	17.04 ± 0.13^c^ (*n* = 433)	19.15 ± 0.11^a^ (*n* = 235)	19.38 ± 0.09^a^ (*n* = 786)
Granum repeat distance (palisade parenchyma) (nm)	19.48 ± 0.17^a^ (*n* =315)	15.47 ± 0.09^b^ (*n* = 513)	16.36 ± 0.15^c^ (*n *= 321)	18.65 ± 0.17^ad^ (*n* = 113)	18.15 ± 0.13^d^ (*n* = 367)
Granum repeat distance (spongy parenchyma) (nm)	19.85 ± 0.14^a^ (*n* =411)	17.18 ± 0.11^b^ (*n* = 386)	19.01 ± 0.20^c^ (*n* = 112)	19.62 ± 0.14^ac^ (*n* = 122)	20.46 ± 0.12^d^ (*n* = 382)

RD values were determined by Fast Fourier Transformation performed on electron microscopic images using ImageJ software. “Ctrl”––control plants analyzed at the same time as “Dry” samples; “Dry”––19-day drought-stressed plants; “R-1 h,” “R-5 h,” and “R-3d”––plants sampled 1 h, 5 h, and 3 days (72 h) after rewatering, respectively. Means and standard error values are provided. Different letters indicate statistically significant differences (*P* ≤ 0.05) as determined by Kruskal–Wallis ANOVA test followed by Dunn's multiple comparison post hoc analysis

Using Mann–Whitney U-test, we did not observe significant change in the number of thylakoid layers present in the grana of control (13.9±3.5, n=59) and drought-stressed (15.8±5.8, n=84) chloroplasts, indicating that granum height was not affected by the treatment.

**Fig. 6:**
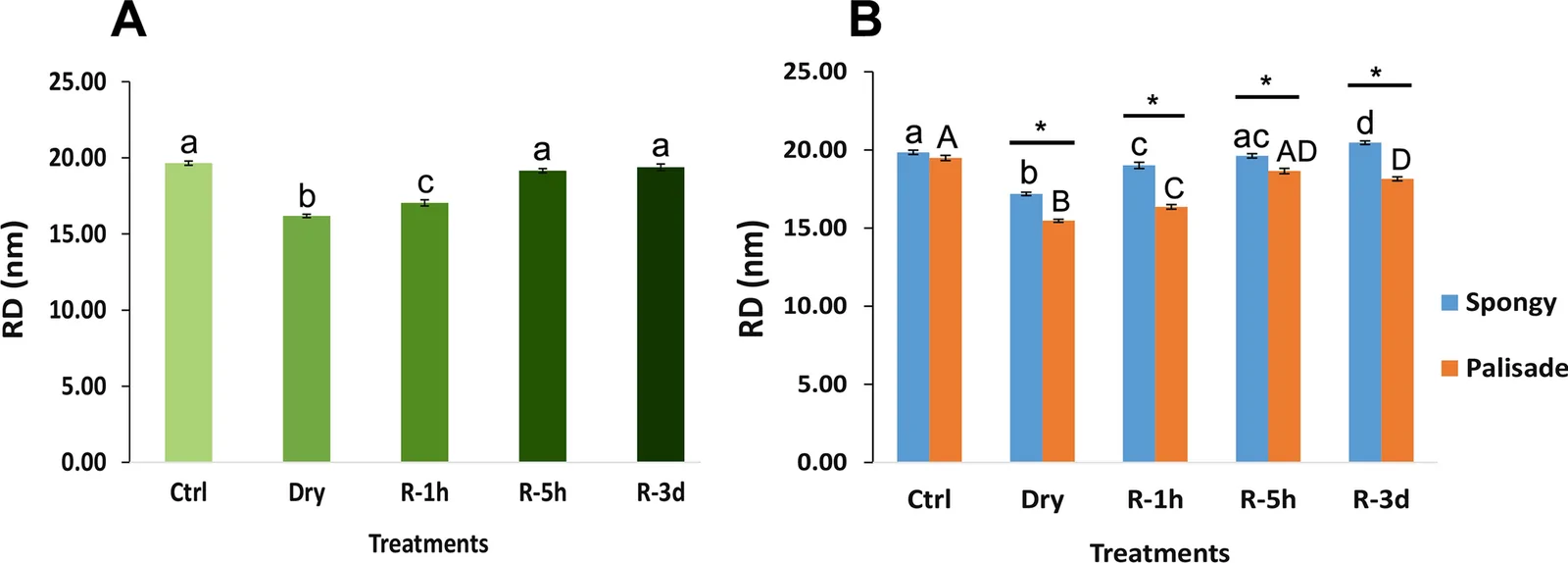
Effect of drought and rewatering on granum repeat distance (RD) values in *J*. *curcas* leaf chloroplasts as assessed by transmission electron microscopy. “Ctrl”––control plants analyzed at the same time as “Dry” samples; “Dry”––19-day drought-stressed plants; “R-1 h,” “R-5 h,” and “R-3d”––plants sampled 1 h, 5 h, and 3 days (72 h) after rewatering, respectively. RD was quantified using ImageJ software by the application of fast Fourier transformation (FFT) to the granum containing image regions. **A** RDs of the mesophyll granum population in the studied plastid sections from leaves under different treatments. Means and standard error values are provided. Different letters within the same group indicate significant differences according to Kruskal–Wallis ANOVA test followed by Dunn’s multiple comparison post hoc test (*P* ≤ 0.001, *n* = 235–914). **B** Differences in granum RD values between the studied chloroplast sections from the spongy and palisade mesophyll regions of the differently treated leaves. Means and standard error values are provided. Asterisks indicate significant differences between the spongy and palisade regions according to a t-test (*P* ≤ 0.001, *n* = 112–513). Different uppercase or lowercase letters indicate statistically significant differences among palisade cell chloroplasts or spongy parenchyma cell chloroplasts across various treatments, respectively, at (*P* ≤ 0.05) as determined by Kruskal–Wallis ANOVA test followed by Dunn’s multiple comparison post hoc analyses (*n* = 112–513)

To characterize granum periodicity, the granum RD values were determined based on FFT analyses of the TEM images of the grana of chloroplasts sampled in the entire mesophyll (Table 2 and Fig. 6A). The RD value decreased significantly in the 19-day drought-stressed *J. curcas* leaves when compared to the control ones (Fig. 6A). RD values started to increase one hour after rewatering and recovered to control values within 5 h of rewatering. Moreover, during our TEM analyses, we observed slight differences in the granum regularity in the chloroplasts located in the spongy and the palisade parenchyma layers under stress conditions and throughout recovery (Table 2 and Fig. 6B). While their RD values were similar under control conditions, the RD values of the grana of the chloroplasts located in the palisade layers decreased more significantly than those of the spongy layers (Fig. 6B). The time required for RD restoration to control levels also differed between the mesophyll tissue layers, occurring within 1 h in the spongy and only after 5 h in the palisade parenchyma layer (Fig. 6B).

**Fig. 7:**
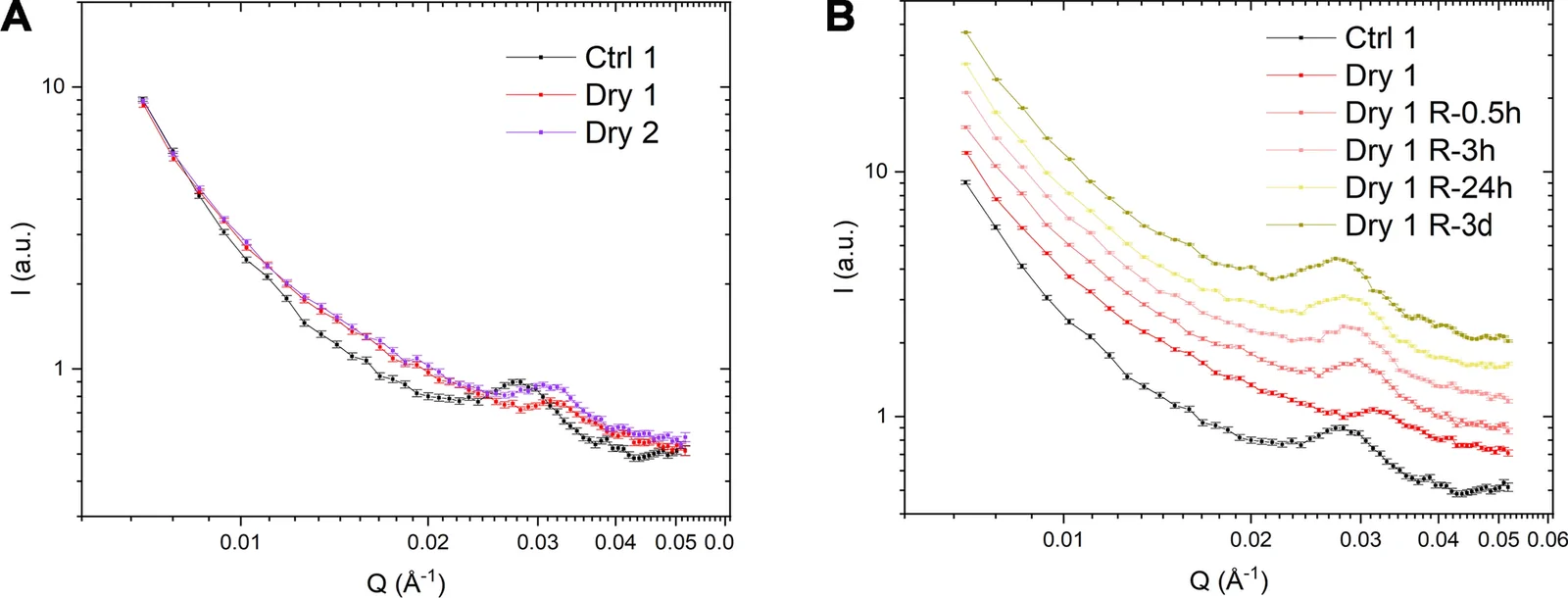
Comparison of the SANS signals obtained from leaf stripes of drought-stressed and rewatered *J. curcas* plants. **A** SANS signal of Control (“Ctrl 1”) and two different 19-day drought-stressed plants (“Dry 1” and “Dry 2”). **B** SANS signal of Control (“Ctrl 1”), drought-stressed (“Dry 1”) and subsequently rewatered plants for 0.5 h, 3 h, 24 h, or 3 days (72 h) (“R-0.5 h,” “R-3 h,” “R-24 h,” or “R-3d”)*.* Curves are vertically shifted for clarity

We compared our RD data obtained on micrographs of chemically fixed and embedded TEM samples with those obtained on leaf strips in vivo by SANS. Comparison of the control and drought-stressed samples revealed significant differences between the SANS profiles––reflecting changes in the organization of the thylakoid membranes’ ultrastructure (Fig. 7A). The drought-stressed plants exhibited somewhat reduced peak intensity, and more stacked thylakoid membranes and thus lower RD values (22.1, 21.7, and 23 nm RD in the control plants and 19.2, 20.1, and 19.9 nm in the drought-stressed plants––Table 3).

**Table 3:** Granum repeat distance (RD) values and correlation peak intensities on the scattering curves of the drought-stressed and rewatered *J*. *curcas* L. leaves as obtained by small-angle neutron scattering (SANS) measurements

	Ctrl1	Ctrl2	Ctrl3	Dry1	Dry1 R-0.5 h	Dry1 R-3 h	Dry1 R-24 h	Dry1 R-3d	Dry2	Dry2 R-12 h	Dry3
RD (nm)	22.1	21.7	23.0	19.2	20.8	21.4	21.8	22.5	19.9	21.3	20.1
a.u [× 10^–3^]	1.69 (8)	1.75 (13)	2.67 (20)	0.62 (8)	1.61 (18)	1.69 (14)	1.97 (7)	2.23 (11)	1.25 (11)	1.72 (11)	1.34 (14)

Abbreviations refer to the following samples: “Ctrl”––control plants analyzed at the same time as “Dry” samples; “Dry”––19-day drought-stressed plants; “R-0.5 h,” “R-3 h,” “R-24 h,” and “R-3d”––plants sampled 0.5 h, 3 h, 24 h, and 3 days (72 h) after rewatering, respectively. The error calculated from the fitting of the Gaussian peaks was in all cases within 0.1 nm

The observed differences between the membrane RD values and correlation peak intensities on the scattering curves of the drought and control samples were almost fully eliminated (from 19.9 to 21.3 nm) within 12 h of the rewatering of the dry plants (Fig. S4A). For a better time-resolution of the rewatering process, we performed multiple time-point measurements during the rewatering of one drought-stressed plant (“Dry 2” plant) (Figs. 7B, S4B). Three days after the rewatering, a full recovery from the drought-induced shrinkage was observed (Table 3), but already 30 min (0.5 h) after rewatering the position of the Bragg peak shifted toward the left (i.e. toward larger RD values) indicating fast recovery (Fig. 7B, Table 3).

It is important to note that even though we observed some natural biological variability in the periodicity of the thylakoid membranes of the control (“Ctrl”) samples, the differences between the control (or 72-h rewatered–– “R-3d”) samples and the “Dry” samples are much more significant (t-test performed on “Ctrl” and “Dry” samples’ RD values indicated significant difference among the two groups). The drought-stressed (“Dry”) samples exhibited scattering peaks with reduced intensity (Table 3) and at a higher momentum transfer value. However, t-test performed on correlation peak intensities of the scattering curves did not reveal statistically significant differences between “Ctrl” and “Dry” samples.

Our previous measurements were performed on non-infiltrated but excised leaf stripes. To check whether excised leaf stripes and intact plants had similar SANS data and to characterize the spatial heterogeneity of the SANS signals within the same leaf, we performed further experiments on intact potted control plants (Fig. S5). The control RD values obtained this way from 3 distinct regions of the same control leaf were not significantly different from each other (20.5, 21.1 and 20.7 nm, for positions 1, 2 and 3, respectively, in Fig. S5) and from the data obtained for control leaf stripes (Table 3).

## Discussion

In the context of climate change, improving our understanding of how drought and post-drought recovery affects the photosynthetic apparatus is particularly relevant, especially across species with contrasting drought strategies. *J. curcas* is a promising biodiesel crop with an effective water retention strategy through stem succulence and other water-saving mechanisms such as early stomatal closure and reduced growth to reduce transpiration area upon drought (Maes et al. 2009; Díaz-López et al. 2012; Sapeta et al. 2013, 2023; Abrar et al. 2020; Della Torre et al. 2021). Consistent with these reports, *J. curcas* maintained high leaf mesophyll water status in our experiment (RWC 88.5%) even at the end of water withdrawal, when soil water content (SWC) had declined to 12% (Table 1).

Our light microscopic observations align with the observed well-preserved water content under drought, as leaf thickness and overall anatomy showed no visible changes (Table 1). However, it was reported that exposure of *J. curcas* seedlings to extremely long drought (90 days) resulted in decreased leaf thickness (Della Torre et al. 2021). Moreover, the leaves of the young seedlings presented in our work had a similar structure but were thinner than those of the 1- and 2-year-old well-watered plants grown under high sunlight exposure in the experimental fields in Brazil (Cao et al. 2023). These differences in leaf thickness likely reflect variation in plant and leaf age, drought duration, and growth conditions (including light intensity).

Light microscopic analyses revealed a very strong separation of the leaf islets by cell columns at the leaf vein regions (Fig. 4). This made it difficult to apply the contrast-enhancement method commonly used for leaf or leaf stripe measurements in SANS (e.g. Ünnep et al. 2014b), which involves mild vacuum infiltration of D₂O prior to measurement. Infiltrating a drought-stressed leaf with D₂O could alter its hydration state, making it unclear whether observed changes reflect the natural drought condition or the effects of infiltration. In our study, the leaves provided sufficient SANS signal without D₂O infiltration, allowing in vivo measurements of drought-induced structural changes. Therefore, the data presented here reliably reflect the natural state of the leaf under stress.

The photosynthetic apparatus of *J. curcas* exhibited resilience to drought, as evidenced by the maintenance of total Chl content (Table 1). However, photosynthetic activity declined (Fig. 1), accompanied by alterations in the composition of the photosynthetic apparatus (Fig. 3). Compositional changes were indicated by a significant reduction in the Chl *a*/b ratio (Table 1). As we did not observe any change in the amount of PSI, the decrease in the Chl *a*/b ratio during drought (Table 1) indicates either a reduction in PSII core complexes or an increase in LHCII levels, as LHCII is richer in Chl b, or both (Chen et al. 2016; Levin et al. 2021; Hu et al. 2023). Indeed, reduction in the absolute amount of PSII core was found: the absolute amount of free LHCII trimers, probably originating from the disassembled PSII supercomplexes, increased but elevated amount of PSII core complexes lacking antenna was not detected (Figs. 3B, S2, S3D) in line with previous observations (Sapeta et al. 2023).

Chl-*a* fluorescence induction kinetics of PSII, revealed a small, yet statistically significant decrease in the *F*_*v*_*/F*_*m*_ ratio, which, qualitatively, could be accounted for the observed increase in *F*_*o*_ after 19 days of water withholding and maintained even until 3 days of recovery (Fig. 1) (Díaz-López et al. 2012; Silva et al. 2012, 2015; Sapeta et al. 2016, 2023). This increase in the *F*_*o*_ level might originate from a partial energetic disconnection of LHCII (Fig. 3B) from PSII and/or from the presence of inactive/degraded PSIIs. An interesting feature of drought-stressed *J. curcas* thylakoids is that despite the overall decrease in PSII supercomplexes, the largest complex (C_2_S_2_M_2_) remained close to control levels (Fig. 3B), suggesting that these complexes are preferentially preserved, possibly in a quenched state. It is interesting to note that in heat-acclimated *A. thaliana*, these forms were also present in higher proportions compared to the ones containing fewer bound antennae, and a fraction of them were inactive (Laihonen et al. 2026).

Changes in the strongly and moderately attached LHCII trimers may also explain the decreased *F*_*v*_*/F*_*m*_ values, because if the amounts of bound LHC-s decrease and that of the trimer and monomer complexes increase, it may increase slightly *F*_*o*_ and thus decrease *F*_*v*_ (Figs. 1, 3). Moreover, an increased pool of free LHCII could contribute to enhanced energy dissipation (*NPQ*), reducing the risk of photo-oxidative damage under stress (Nicol & Croce 2021). In agreement with previous reports (Lima Neto et al. 2017; Silva et al. 2012, 2018; Sapeta et al. 2016, 2023), *NPQ* increased drastically during drought (Fig. 1). Such dynamic channeling of excess light energy toward *NPQ* is often observed in drought-tolerant plant species and also already described for *J. curcas* under drought conditions (Sapeta et al. 2023). Overall, these photosynthetic responses align with the established drought physiology of *J. curcas* (Sapeta et al. 2013, 2016, 2023; Lima Neto et al. 2017).

Drought stress significantly diminished the (−)675 nm CD band (Fig. 2), which indicates a perturbation of the stacking interactions; data consistent with densely stacked granum thylakoid membrane structure observed by TEM and SANS data (Figs. 5 and 7, S4 and Tables 2, 3). The exact physical mechanism of these drought-induced reversible changes in the chiral macro-organization of the complexes is not known. However, it is interesting to note that similar hydration dependent variations of the circularly polarized luminescence signal (CPL) have been observed in intact pea leaves (Gussakovsky et al. 2002). Similarly to CD, CPL of intact plant thylakoid membranes and LHCII aggregates predominantly originates from chiral macro-arrays of the protein complexes (Garab & van Amerongen 2009). Similar, albeit more severe changes were observed in pea leaves exposed to drought-stress: it was shown that in addition to a more substantial reduction in the psi-type CD bands, the CD_484-462_ excitonic band, attributed to LHCII trimers (Dobrikova et al. 2003) was also diminished (Pandey et al. 2023).

Under drought stress, *J. curcas* chloroplast ultrastructure was overall well retained (Fig. 5 and Table 2) but drought-induced alterations were observed, such as the absence of starch (Fig. 5C–D). The latter being consistent with reported reductions in leaf starch content under drought (Díaz-López et al. 2012; Hembrom et al. 2025).

Interestingly, despite the well-retained RWC (Table 1) and chloroplast ultrastructure (Fig. 5C–D) under drought, we detected changes indicative of altered chloroplast water or ion homeostasis. These included significant reduction in chloroplast size, and decreased granum repeat distance (RD) values, reflecting more densely stacked thylakoid membranes that were consistently observed by both TEM and SANS (Tables 2 and 3). Similar preservation of plastid structure under drought has been reported in *Ctenanthe setosa* (Hembrom et al. 2025) and *Brassica napus* (Zhu et al. 2021). *C. setosa*, a drought-tolerant species, also exhibited decreased RD values under drought that recovered upon rewatering (Hembrom et al. 2025), suggesting that reduced RD may represent a reversible acclimatory response to drought. In contrast, swelling of the thylakoid lumen has been described in drought-stressed sugarcane (Zhang et al. 2015, 2020). Swelling of lumen may reflect more advanced or irreversible membrane perturbation, potentially depending on species-specific tolerance or stress severity. Therefore, differences among studies likely relate to variation in drought tolerance, genotype, and treatment duration, and would require further investigations.

It is worth mentioning that *Ctenanthe setosa* has better preserved its photosynthetic activity (*F*_*v*_*/F*_*m*_) as well as thylakoid protein composition even after 40–49 days of drought stress when compared to *J. curcas* in this experiment, but had a much slower recovery of the granum RD values happening only 10 h after rewatering (Hembrom et al. 2025). The RD changes were in the same order of magnitude in both species (1–3 nm shrinkage upon drought followed by recovery upon rewatering). However, due to species-specific differences in drought tolerance (moderate leaf- and stem succulence observed in *C. setosa* and *J. curcas*, respectively), root architecture, in the soil types used for their cultivation, the different lengths of the treatments and age of plants, quantitative comparisons are not adequate between the two experiments and species. However, we might speculate that better thylakoid preservation in *Ctenanthe* comes at a cost of slower and reduced structural flexibility even during recovery (Hembrom et al. 2025), while the very fast structural recovery of the RD values in *Jatropha* might be a prerequisite for the more extensive repair and restoration processes associated with the recovery of the photosynthetic activity after drought stress. Such processes might include cleavage of damaged proteins and other thylakoid components and insertion of newly synthesized components into the thylakoid, processes which are probably facilitated in thylakoids with increased RD values.

Recent data presented sophisticated temporal and spatial, mainly vertical dynamic regulatory mechanisms in granum structure and function, revealing initial shrinkage of granum RD values upon light as a mechanism influencing cyclic/linear electron transport ratio and thus photoprotection by affecting the reorganization of massive complexes of the electron transport chain (Wójtowicz et al. 2025). Thus, minor alterations in granum RD values might play an important regulatory role in photosynthesis. Unfortunately, no generally accepted and functional model is available to further resolve RD values and allow the deduction of detailed structural parameters of the thylakoids from SANS data of non-infiltrated intact leaves with the resolution, q range and statistics available for our non-infiltrated intact leaves. Existing models have strong limitations and were only applied on infiltrated leaves and thylakoid membranes (Wójtowicz et al. 2025). Thus, we would prefer not to speculate about these values. Concerning the observed changes, the thickness of the two lipid membrane bilayers is probably not much altered by drought stress, which might rather influence the width of the interthylakoidal space (also called stromal gap or partition gap) or more importantly that of the lumen (intrathylakoidal space), both aqueous phases having important roles in maintaining granum structure and function.

SANS provides spatially averaged data on all grana present in the leaf area within the neutron beam under in vivo conditions (Nagy & Garab 2021) and is thus very useful to monitor changes during recovery of these parameters after rewatering (Hembrom et al. 2025). We have not observed any difference in the number of granum layers of control and drought-stressed chloroplasts, therefore, the observed reduced Bragg peak intensities under drought stress (Fig. 7 and Table 3) may be a result of either the reduced contrast between the membranes and the surrounding liquid phase and/or the reduced order in the system due to the potential bending stress exerted by the suboptimal amount of H_2_O between the adjacent layers (Fehér et al. 2025). When talking about the intensity of the scattering peaks, it is important to note that the presented scattering curves are not in absolute scale, since the total thickness of the measured leaf segments could not be precisely determined in the SANS laboratory. However, we can safely assume that the difference between the total thickness of the samples in the freestanding-leaf measurements was in the range of tens of percents since the scattering curves are well overlapping both in the low and in the high-Q regions (i.e. far from the position of the thylakoid membrane related Bragg-peak); also, light microscopy revealed unchanged leaf thickness across treatments (Fig. 4).

The fact that we obtained slightly larger RD values for all samples by SANS than by TEM (Tables 2 and 3) is in line with previous observations (Ünnep et al. 2014a; Hembrom et al. 2025) and is probably related to the shrinkage of the granum membranes in chemically fixed and dehydrated biological samples. Despite the differences between SANS and TEM methods, the same tendencies were observed making the findings very robust.

As *J. curcas* has thick leaves with well-distinguishable heterogeneous mesophyll organization also in TEM, in this work we were able to distinguish between the different granum subpopulations within a leaf, i.e. those present in the palisade or spongy parenchyma chloroplasts (Table 2 and Fig. 6). These differences are not visible for SANS (as it collects spatially averaged signal from all grana present in the leaf) but could be detected by TEM. RD values of grana in palisade and spongy parenchyma chloroplasts were similar in control plants, but drought stress resulted in significantly lower RD values in palisade parenchyma plastids than in those in the spongy layer. Through recovery, this stronger negative effect and slower recovery of the grana in the palisade parenchyma could be clearly observed (Table 2 and Fig. 6). This might be related to the different anatomy of the two layers, and slower rehydration of the adaxial palisade layer cells upon rewatering in contrast with the spongy parenchyma surrounded by large intercellular cavities filled with water vapor via relaunched transpiration in the plants. Differences between spongy and palisade parenchyma chloroplast number, size or even thylakoid stacking are well-known from literature data (Schwarz 1988; Adolfsson et al. 2015), however, to the best of our knowledge, this is the first time that differences are reported in the drought sensitivity and recovery speed of their granum RD values.

Ultrastructural recovery of the chloroplasts after rewatering was remarkably rapid. Granum organization recovered and chloroplast length and length-to-width ratio were restored within 0.5–1 h (Tables 2 and 3). The ( −)675 nm psi-type CD band almost fully recovered after 5 h of rewatering (Fig. 2), further indicating rapid reorganization of the thylakoid membrane system. The observed decline in plastid size and shape that occurred 3 days after rewatering might be associated with plastid division, promoting increased photosynthetic capacity to boost growth restart after stress relief. Functional and compositional recovery of the photosynthetic apparatus occurred more gradually. Most physiological and biochemical parameters, such as photosynthetic activity (*F*_*v*_/*F*_*m*_ and *NPQ*) (Fig. 1), the Chl *a*/b ratio (Table 1), and several Chl-protein complexes (Fig. 3) recovered between 3 and 21 days of rewatering.

Overall, our findings indicate that the rapid reorganization of granum ultrastructure reflects a remarkable flexibility of thylakoid architecture, allowing *Jatropha curcas* to withstand long-periods of drought and resume photosynthetic function and growth once water is available.

## Data Availability

The corresponding author will provide the data supporting the findings of this study upon reasonable request.
